# The proportion of South Africans living within 60 and120 minutes of a percutaneous coronary interventionfacility

**DOI:** 10.5830/CVJA-2018-004

**Published:** 2018

**Authors:** Stassen Willem, Wallis Lee, Vincent-Lambert Craig, Castren Maaret, Kurland Lisa

**Affiliations:** Department of Clinical Research and Education, Karolinska Institute, Stockholm, Sweden; and Division of Emergency Medicine, Stellenbosch University, Stellenbosch, South Africa; Division of Emergency Medicine, Stellenbosch University, Stellenbosch, South Africa; Department of Emergency Medical Care, University of Johannesburg, Johannesburg, South Africa; Department of Clinical Research and Education, Karolinska Institute, Stockholm, Sweden; and Department of Emergency Medicine and Services, Helsinki University, Helsinki, Finland; Department of Clinical Research and Education, Karolinska Institute, Sweden; and Department of Medical Sciences, Örebro University, Örebro, Sweden

**Keywords:** myocardial infarction, healthcare disparities, percutaneous coronary intervention,, South Africa

## Abstract

**Introduction:**

Timely reperfusion, preferably via percutaneous coronary intervention (PCI) following myocardial infarction, improves mortality rates. Emergency medical services play a pivotal role in recognising and transporting patients with ST-elevation myocardial infarction directly to a PCI facility to avoid delays to reperfusion. Access to PCI is, in part, dependant on the geographic distribution of patients around PCI facilities. The aim of this study was to determine the proportion of South Africans living within 60 and 120 minutes of a PCI facility.

**Methods:**

PCI facility and population data were subjected to proximity analysis to determine the average drive times from municipal ward centroids to PCI facilities for each province in South Africa. Thereafter, the population of each ward living within 60 and 120 minutes of a PCI facility was extrapolated.

**Results:**

Approximately 53.8 and 71.53% of the South African population live within 60 and 120 minutes of a PCI facility. The median (IQR, range) drive times and distances to a PCI facility are 100 minutes (120.4 min, 0.7–751.8) across 123.6 km (157.6 km, 0.3–940.8).

**Conclusion:**

Based on the proximity of South Africans to PCI facilities, it seems possible that most patients could receive timely PCI within 120 minutes of first medical contact. However, this may be unlikely for some due to a lack of medical insurance, under-developed referral networks or other system delays. Coronary care networks should be developed based on the proximity of communities to 12-lead ECG and reperfusion therapies (such as PCI facilities). Public and private healthcare partnerships should be fortified to allow for patients without medical insurance to have equal accesses to PCI facilities.

## Introduction

Ischaemic heart disease (IHD) is projected to double in incidence within sub-Saharan Africa within the next few years.^1^,^2^ For a variety of reasons, African healthcare services may not be prepared to manage these lifestyle diseases.^3^ ST-elevation myocardial infarction (STEMI), a time-sensitive consequence of cardiovascular disease progression, should be managed emergently in order to decrease morbidity and mortality rates.^4^-^8^

According to the American and South African Heart Associations, percutaneous coronary intervention (PCI) is the preferred method of reperfusion for STEMI, and should be performed within 120 minutes of first medical contact.^4^,^9^,^10^ Despite this recommendation, only 61.3% of patients who present with STEMI in South Africa receive reperfusion via PCI within 24 hours. In 34.8% of patients, the indication for PCI was failed thrombolysis.^11^

For patients who cannot reach a PCI facility within 120 minutes, it is recommended that reperfusion be obtained by means of thrombolytic therapy within 30 minutes of first medical contact. This could be initiated by pre-hospital emergency care providers.^4^ Delayed reperfusion can be attributed to: late patient presentation, protracted pre-hospital response and scene times, delays in 12-lead ECG acquisition and STEMI diagnosis, transport to non-PCI facilities requiring secondary interfacility transfer, and PCI preparation time.^12^-^14^

To minimise these delays, it has been suggested that 12-lead ECG and STEMI diagnosis should become standard practice in the pre-hospital setting. This would allow for patients to be transported directly to a PCI facility.^4^ However, as outcome is linked to the time to reperfusion, the outcome benefit gained of initial transport to a PCI facility may be offset by protracted transport times to such facilities. The geographic distribution of patients and PCI facilities and their relative proximity will therefore impact on the feasibility of these recommendations, and the successful development and implementation of regional coronary care networks for patients with STEMI.

The aim of this study was to determine the proportion of South Africans who live within 60 and 120 minutes of a PCI facility. To this end, we determined the driving times and distances from each municipal ward to the closest PCI facility. This can be used as a measure of access and as a guide for future development of coronary care and referral networks.

## Methods

We assessed timely access to PCI facilities by a series of geospatial analyses. Firstly, we determined the driving times and distances to the closest (private and/or public) PCI facility of each of the municipal wards within South Africa. Hereafter, we determined the proportion of the South African population who live within 60 and 120 minutes of these facilities, based on the average driving times. We purposefully selected these time frames as they are in line with local and international PCI reperfusion guidelines.^4^,^10^

PCI facility availability data from a previously published cross-sectional study were utilised.^15^ We plotted public and private PCI facilities in turn, using the physical address of each. From here we used ArcGIS 10 and ArcGIS Online (Esri, California, United States) to plot a 60- and 120-minute drive-time polygon around each of the PCI facilities. ArcGIS calculates the drivetime polygons around created points (PCI facilities, in this case) that can be accessed within a specified time of travel from that point. These drive times are calculated using predicted typical traffic trends. Typical traffic trends for each road are determined within ArcGIS by averaging a week’s real-time travel speeds in five-minute intervals.

Using ArcGIS, a join was created between the current South African ward boundary lines and the 2011 population census data.^16^ Ward (district)-level data were used as this is the smallest geographical area available with population data, which improves accuracy of results. Ward-level data were not available for the 2016 community survey. The mathematical mid-point (centroid) of each ward was calculated and the population was added to this point on the map datasets.

Proximity analysis was used to determine the projected driving time from each ward centroid to the closest PCI facility in all provinces. These driving times were again calculated based on the typical traffic trends for each area. These data are presented descriptively. Medians and interquartile ranges are reported as the data showed heterogeneity between provinces.

Using the drive-time polygons and the ‘Select by Location’ feature of ArcGIS 10, it was possible to extract those wards whose centres fell within the 60- and 120-minute drive-time polygons.^17^ Integrity of the data was ensured by performing a series of manual verifications. We extracted the specific wards and their populations that fell within these polygons to determine the population who live within these referral areas.

Ethical approval was obtained from the Human Research Ethics Committee of the University of Stellenbosch (HREC Ref Nr: M14/07/027).

## Results

**Fig. 1:**
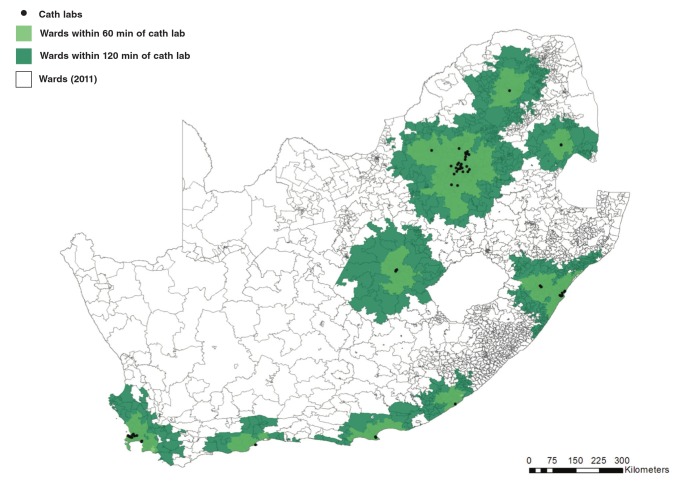
Drive-time polygons and wards within 60 and 120 minutes of PCI facilities (ArcGIS 10, Esri, California, United States)

PCI facilities are concentrated around major cities and along the coastal areas of South Africa. Wards within the 60- and 120-minute drive-time polygons to PCI facilities are presented in Fig. 1.

Table 1 displays the driving distances to PCI facilities in South Africa. The median driving distance to the closest PCI facility nationally is 123.6 km (IQR: 157.6 km). The Northern Cape has the longest driving distance to a PCI facility, of 940.8 km, while the shortest distance is 0.3 km in the Eastern Cape and the Free State provinces, jointly. The median driving distance to the closest public PCI facility (Table 2) is 100 km (IQR: 157.6 km), while the shortest median driving distances are in the Free State and the Gauteng provinces, jointly (0.9 km), and the furthest is in the Northern Cape (1 085 km).

**Table 1:** Driving distances to a PCI facility (public or private) in South Africa

*Province*	*Minimum drive distance (km)*	*Median drive distance (km) (IQR)*	*Maximum drive distance (km)*
Gauteng	0.5	15.1 (15.9)	71.5
Western Cape	0.8	52.9 (110.3)	363
Northern Cape	155.5	406.9 (305.4)	940.8
Eastern Cape	0.3	206.9 (157.2)	395
North West	1.1	144 (162.6)	573.3
KwaZulu-Natal	1.6	137.5 (166.6)	413.8
Free State	0.3	140.1 (118.1)	278
Mpumalanga	2.0	109.7 (51.7)	313.1
Limpopo	2.3	132 (73.9)	342.9
South Africa	0.3	123.6 (157.6)	940.8

**Table 2:** Driving distances to a public PCI facility in South Africa

*Province*	*Minimum drive distance (km)*	*Median drive distance (km) (IQR)*	*Maximum drive distance (km)*
Gauteng	0.9	28.7 (28.5)	90.6
Western Cape	1.4	93 (227.8)	495.6
Northern Cape	155.5	406.8 (311.6)	1085.8
Eastern Cape	2.4	302.3 (140.4)	551.7
North West	7.0	172.3 (173.9)	653.7
KwaZulu-Natal	2.4	146.3 (173.8)	434.8
Free State	0.9	158.3 (107.8)	301.8
Mpumalanga	7.4	125.5 (52.5)	320.4
Limpopo	77.0	289.2 (158.1)	607.8
South Africa	0.9	170.7 (22.35)	1085.8

Table 3 displays the driving times to the PCI facilities in South Africa. The longest drive to PCI is in the Northern Cape at 751 minutes while the shortest drive is in the Free State province (0.7 minutes). Nationally, the median driving time to PCI is 100 minutes (IQR: 120.4). The closest public PCI facility (Table 4) is a median of 123.7 minutes (IQR 164.1) away. The shortest time to the closest public PCI facility is in KwaZulu-Natal (1.5 minutes away) while the furthest is in the Northern Cape (900.1 minutes away).

**Table 3:** Driving times to a PCI facility (public or private) in South Africa

*Province*	*Minimum drive time (min)*	*Median drive time (min) (IQR)*	*Maximum drive time (min)*
Gauteng	0.8	18.3 (13.8)	59.6
Western Cape	1.6	43.8 (79.3)	277.9
Northern Cape	111.7	300.4 (640.1)	751.8
Eastern Cape	0.8	164 (137.5)	318.6
North West	3.1	115.4 (117.3)	453.6
KwaZulu-Natal	3.0	109.8 (133.6)	345.1
Free State	0.7	103.1 (79.9)	227.0
Mpumalanga	4.2	94.4 (54.6)	249.2
Limpopo	3.5	114.1 (63.2)	344.3
South Africa	0.7	100 (120.4)	751.8

**Table 4:** Driving times to a public PCI facility in South Africa

*Province*	*Minimum drive time (min)*	*Median drive time (min) (IQR)*	*Maximum drive time (min)*
Gauteng	1.7	29.1 (20.6)	68.3
Western Cape	3.0	77.8 (160.4)	328.7
Northern Cape	105.4	298.1 (210.9)	900.1
Eastern Cape	4.3	238.6 (127.2)	432.6
North West	10.5	134.2 (125.8)	486.7
KwaZulu-Natal	1.5	90.9 (108)	270.2
Free State	1.7	112.5 (79.1)	242.9
Mpumalanga	12.6	102.6 (53.4)	257.8
Limpopo	80.8	230 (88.3)	515.2
South Africa	1.5	123.7 (164.1)	900.1

Just over half of the population (53.8%) of South Africa live within 60 minutes of a PCI facility while 71.53% of the country’s population can reach a PCI facility within two hours (Table 5). Practically all inhabitants of the Gauteng province live within 60 minutes of PCI while 2.5% of the Northern Cape’s inhabitants are within two hours of the closest PCI facility, whether public or privately owned. When only considering public PCI facilities (Table 6), only 47.8 and 63% of the population can access these facilities within 60 and 120 minutes, respectively.

**Table 5:** Proportion of South African population living within 60 and 120 minutes of a public or private PCI facility

*Province*	*PCI within 60 minutes n (% per province)*	*PCI within 120 minutes n (% per province)*
Gauteng	12.27 mil (99.7)	12.3 mil (100)
Western Cape	4.44 mil (76.1)	5.1 mil (87.6)
Northern Cape	0 (0)	29 000 (2.5)
Eastern Cape	1.96 mil (29.9)	2.68 mil (40.8)
North West	1.28 mil (36.4)	2.13 mil (60.6)
KwaZulu-Natal	4.89 mil (47.6)	6.64 mil (64.7)
Free State	0.99 mil (36.4)	1.9 mil (69.3)
Mpumalanga	0.95 mil (23.5)	3.19 mil (78.9)
Limpopo	1.06 mil (19.8)	3.04 mil (56.3)
Total, n (% SA)	27.86 mil (53.8)	37.0 mil (71.5)

mil: million.

**Table 6:** Proportion of South African population living within 60 and 120 minutes of a public PCI facility

*Province*	*PCI within 60 minutes n (% per province)*	*PCI within 120 minutes n (% per province)*
Gauteng	12.27 mil (99.7)	12.27 mil (99.7)
Western Cape	4.19 mil (71.9)	4.78 mil (82)
Northern Cape	0 (0)	0 (0)
Eastern Cape	1.22 mil (18.6)	1.48 mil (22.6)
North West	0.66 mil (18.8)	1.93 mil (55)
KwaZulu-Natal	4.78 mil (46.6)	6.72 mil (65.4)
Free State	0.82 mil (29.9)	1.81 mil (65.9)
Mpumalanga	0.73 mil (18.1)	3.13 mil (77.5)
Limpopo	6 000 (0.1)	0.26 mil (4.9)
Total, n (% SA)	24.6 mil (47.8)	32.6 mil (63.0)

mil: million.

## Discussion

Approximately 53.8 and 71.5% of the South African population live within 60 and 120 minutes of a PCI facility, respectively. The median distance from a PCI facility nationally is 123.6 km while the median driving time to a PCI facility is 100 minutes.

In the United States, 79% of the adult population live within one hour of a PCI facility. For those living further away, 74% would be able to access a PCI facility with an additional drive of less than 30 minutes,^18^ well within the recommendations.^4^ There is currently one PCI facility for every 887 096 people in South Africa,^15^ which would be sufficient if every patient could access this facility within 120 minutes of first medical contact.19 This is however only achievable for 71.53% of the population. More PCI facilities are therefore needed.

Despite living in close proximity to PCI facilities, only 61.3% of STEMI patients receive PCI within 24 hours.^11^ This might suggest that larger system problems contribute to further delays.^12^-^14^

Access is not simply a product of proximity, but also of socio-economic status and other demographic factors.^3^,^15^,^20^-^22^ Low-income patients living in rural areas and those without medical insurance experience the greatest barriers to accessing healthcare services.^20^ In South Africa, 77% of all the PCI facilities are owned by the private healthcare sector and can therefore only be utilised by 18.1% of the population,15 unless upfront payment of up to $3500 (~R50 000) is made.^15^

When we consider this, the proportion of South Africans who can access PCI within 60 (53.8%) and 120 minutes (71.53%) is an over-estimation, as access is often limited to insurance status. In South Africa, the median driving times for uninsured patients to the closest public PCI facility are 123.7 minutes across 170.7 km, while only 47.8% and 63.0% of the population can access these facilities in 60 and 120 minutes respectively. It is recommended that patients who experience symptoms of myocardial infarction be transported to hospital via emergency medical services (EMS) so that suitably qualified pre-hospital emergency care providers can start treatment and manage any complications that might arise.^4^ Locally, the majority of patients seem to be transported privately.^12^-^13^ Reasons for this include unfamiliarity with emergency numbers, poor and unreliable response times of EMS, or lack of understanding of the value of EMS use in myocardial infarction.^12^

Mistrust in the EMS is not unfounded as up to 95% of urban and 68% of rural high-acuity responses are not serviced within 15 and 40 minutes, respectively.^23^ One study has shown that in 16.7% of responses, public ambulances may take more than 12 hours to arrive in certain rural areas of the country.^24^ In Africa, EMS systems are often informal with unreliable coverage.^25^ Ambulance transport may not always be feasible for Africans with STEMI,^25^ and pre-hospital delays can have significant effects on the reperfusion times of patients regardless of their proximity to a PCI facility.^26^

For patients who cannot reach a PCI facility timeously, pre-hospital thrombolysis is recommended.^4^ At present, only emergency care practitioners who hold a bachelor degree qualification can administer pre-hospital thrombolysis in South Africa.^27^,^28^ Recommendations are that, should pre-hospital thrombolysis be considered, it should be performed within a well-developed coronary care network that can manage failed thrombolysis and other complications.^28^ We found that most PCI facilities are concentrated in the urban areas. Unfortunately, within our setting, there is misdistribution of advanced life support (ALS) paramedics, with most practicing in urban areas.^29^ Steps should be taken to promote recruitment, deployment and retention of paramedics in these rural areas.

The utilisation of helicopter emergency medical services (HEMS) has been suggested to improve the reperfusion times30 of STEMIs and to deliver ALS care to patients in rural areas.^31^ Considering the shortage of ground-based ALS, HEMS may be a feasible option for delivering pre-hospital thrombolysis to many remote communities, however, the benefit of this resource should be offset by its cost burden in the context of low- and middle-income countries such as South Africa.^31^ Further to this, activation of HEMS should be subject to confirmed STEMI diagnosis by on-scene providers. Until now, 12-lead ECG acquisition and interpretation has been a skill reserved only for ALS providers.^27^

Pre-hospital 12-lead ECG acquisition and interpretation has also been extended to the mid-level EMS worker (emergency care technicians), which may expedite STEMI diagnosis and decrease reperfusion times.^32^ Upskilling in this regard may be required, as studies have shown that a delay in reperfusion may occur when inexperienced providers doubt the ECG diagnosis.^12^ Pre-hospital 12-lead ECG telemetry has been applied in developed countries and may be used to expedite reperfusion.^33^ A randomised, controlled trial was undertaken in 2016 in South Africa to determine the application of 12-lead ECG telemetry in this context (pers commun).

## Limitations

This study has some important limitations. Drive-time polygons were generated based on typical (average) driving times and traffic conditions. Response and ambulance scene times, which may prolong the pre-hospital time, were not taken into consideration. In addition, for patients not utilising ambulance transport to hospital, time to access private or public transport was not taken into consideration.

Census data from 2011 was used as the 2016 community survey data from Statistics South Africa provide population data only up to municipal level, as the sample size does not allow for analysis at ward level.

Again it is essential to reiterate that expressing access in this study assumes that any patient can be treated at any facility. However, in practice most facilities (77%) are only accessible to the 18% of patients with medical insurance.^15^,^34^

Using epidemiological and geospatial data, formal referral networks and guidelines could be developed that are contextual to each specific region within South Africa (and Africa), and that take into consideration the specific resources available and the proximity to these resources. In addition, it is essential to establish what the capacity and role of EMS is within the African context to improve reperfusion times for patients suffering myocardial infarctions.

## Conclusion

Up to 72% of South Africans live within two hours of a PCI facility, but timely access may not be possible because of insurance status or other system delays. The incidence of ischaemic heart disease is on the increase in South Africa. In order to prepare for this epidemiological transition, there is a pressing need to develop coronary care networks to provide emergency care for these patients. Development of coronary care networks should be prioritised by policy makers and tailored to the specific proximity to 12-lead ECG, thrombolysis or PCI of each community.
